# An empirical analysis of safety behaviour: A study in MRO business in Indonesia

**DOI:** 10.1016/j.heliyon.2021.e06122

**Published:** 2021-02-15

**Authors:** Erman Noor Adi, Anis Eliyana, Hamidah

**Affiliations:** a Universitas Negeri Jakarta, Indonesia; b University Airlangga, Indonesia

**Keywords:** Safety behaviour, Safety leadership, Safety communication, Safety commitment, Safety climate, Safe working environment

## Abstract

This study aims to investigate employee safety behaviour by relating it to safety leadership, safety communication, safety commitment, and safety climate. This research was conducted at PT GMF AeroAsia Tbk in the Cengkareng home base, and multibase areas of Kalimantan, Bali & Nusa Tenggara, Sumatra, Sulawesi & East, and Java. The study began in early September 2019 until the end of March 2020, using a quantitative and explanatory design approach through testing hypotheses to examine the nature of relationships and influences between variables. The population of 2,400 employees with a sample of 342 respondents. The sample distribution uses proportionate cluster random sampling. Model testing and data processing using Structural Equation Modelling (SEM). The data was then analysed using AMOS 22 statistical software. Work accidents at the largest MRO (Maintenance Repair and Overhaul) companies were mainly caused by safety behaviour. Test results from several variables of this study can help the managerial team make an effective approach to improve safety behaviour in the workplace.

## 1 Introduction

Indonesia is one of the regions with the highest fleet growth (7.4%) among other countries. To ensure the operation and safety of aircraft, regular maintenance and periodic repairs are needed by aircraft maintenance organizations, known as MROs (Maintenance, Repair & Overhaul). The growth of the aircraft business inevitably has an impact on the need for MRO because maintenance becomes the absolute necessity of every fleet. This condition makes MRO a strategic industry in aviation. The MRO industry also has an economic impact in the form of adding foreign exchange and increasing local content. In addition, the growth of the MRO industry also encourages the creation of high-tech jobs, as well as creating a multiplier effect on supporting industries.

In Maintenance, Repair and Overhaul (MRO) Fundamentals and Strategies (Rodrigues Vieira and Loures, 2016), Kannisson stated that maintenance refers to a process of confirming that a system continues to perform its standard function at a good reliability and security level. Whereas Viles emphasized that the purpose of maintenance is not only to minimize time to do reparation but also to increase the reliability of the product, as well as to gain relevant information for analysis.

With vital functions in the aviation world, the MRO industry continuously has challenges and needs efforts to overcome them so that the industry is able to survive, grow, and develop. One effort that continues to be done is to overcome the problem of accidents in the workplace that often cause harm to individuals and companies. The main cause of decreased individual productivity is workplace accidents (Spurgeon et al., 1997). Work accidents occur due to many factors. One factor that contributes to the incidence of workplace accidents is the human factor.

At present, PT GMF AeroAsia Tbk is the most outstanding and largest MRO company in Indonesia, as well as number 4 in Asia and number 9 in the world. GMF employs approximately 5,000 employees based in Jakarta and spread in 47 airports at home and abroad. GMF provides integrated solutions to 180 customers spread across 5 continents in more than 60 countries in the world. GMF is engaged in aircraft maintenance, aircraft engine and aircraft components, as well as off-plane turbine engines. Maintenance, repairs, and overhauls often use hazardous and toxic substances (B3) that have the potential to cause poisoning, explosion, and fire. Facilities and infrastructure in the form of equipment and machinery also have the risk of causing various work accidents. GMF made various efforts to increase understanding of safety improvement through discussion programs, prints & running text, SMS (Safety Management System), briefing sheets, audio, videos, and websites, and PENITY magazine which has been published since October 2018 and has a specific purpose of conveying knowledge and information about safety to all GMF personnel. The information contained is relevant to the situation faced by individuals in the company.

The quality of work safety at the company is crucial to improve safety behaviour. To build the desired work safety environment properly, it takes the role and contribution of both leaders and employees, which eventually influence the safety behaviour of each individual in a company.

Leaders who underline the urgency of safety among company members will increase safety climate which then prompts workers to adopt behaviours that also prioritize the value of work safety (safety behaviour) (MartínezCórcoles, Gracia, Tomas and Peiró, 2011). According to (Cooper, 2015) safety leadership is a process of leaders in forming teams, ensuring that teams are involved in encouraging safety values and maintaining the team to achieve organizational safety goals. According to Hair et al. (1995) in (Lu and Yang, 2010), a person can be said to be a safety leadership if he/she has fulfilled three aspects namely safety motivation, safety policy and safety concern.

To socialize the importance of work safety values, good communication process must be built. Communication helps workers understand contradictory targets, reduce uncertainty, and serve as a consensus basis on how to work appropriately (D. Zohar and Tenne-Gazit, 2008). Safety communication has an educational function related to unclear situational hazards and a changing work environment and work assignments (Cigularov et al., 2010). It may also reduce potential risks such as work accidents and other negative impacts related to work.

Furthermore, to apply safety values a commitment is needed. A study conducted by Laura S. Fruhen, Griffin and Andrei (2019) measured safety commitment through five aspects of management actions namely decision making, managerial policy, active involvement, and communication with the workforce. In fact, in some safety literature safety commitment is emphasized as an important influence on organizational safety and is a major component of the safety climate or safety culture.

In D. Zohar and Luria's (2003) study, safety climate is explained as a priority for work safety and this perception in turn predicts the behaviour that will be seen (Neal and Griffin, 2009). Safety climate according to Flin et al. (2006) is the perception of company members about the work environment and work safety regulations that are in it. Furthermore, safety climate has a direct influence on safety behaviour (Buttimer et al., 2008). Safety climate is often associated with the occurrence of work accidents in companies because safety climate may possibly affect the number of incidents in the workplace (Liu et al., 2015). Positive safety perceptions by workers will affect worker safety behaviour (Seo, 2005). Moreover, research from Zhou et al. (2008) proves that this is the most effective factor for improving safety behaviour.

Based on this description, this study aims to prove the effect of safety leadership, safety communication and safety commitment on safety climate, the effect of safety leadership, safety communication, safety commitment and safety climate on safety behaviour, and the last is the mediating effect of safety climate on safety leadership, safety communication and safety commitment to safety behaviour.

## 2 Theories

### 2.1 Safety behaviour

Behaviour is simply an actual activity or behaviour that someone has demonstrated in relation to other individuals and the world around them. Employees operate as part of the company, based on company policies. The term safety behaviour itself is an activity conducted by individuals within an entity related to protection (He et al., 2019). Safety behaviour as a primary mark of safety performance has many benefits over lagging factors, such as injury and death. The data are more often to have a regular distribution, so it is easier to interpret the relationship with the antecedents, more reliable, and more useful for safety evaluation and intervention. In addition, safety behaviour is inseparable with safety performance, and is affected by multiple processes, according to Xue et al. (2020). Moreover, it is classified into two main categories: safety compliance and safety participation (Amponsah-Tawaih and Adu, 2016). Compliance with safety relates to acts regarding safety in roles, such as compliance with safety regulations and adherence to safety instructions. Safety participation is safety-related behaviour that does not play a role or is voluntary, such as willingness to support co-workers, to engage regularly in safety training programs, and to make safety recommendations. Based on the above definition, it can be synthesized that safety behaviour refers to employee safety activity in the workplace that is expressed by employee actions to build and enhance safety at work environment.

### 2.2 Safety leadership

Safety leadership is defined as a complex construction that reflects the value of safety, shown through attitudes and activities that directly affect workplace safety (Schwatka et al., 2019). Meanwhile, according to Oah et al. (2018), safety leadership is an interaction process between leaders and members in which leaders can guide members to accomplish organizational safety targets hence supervisor or leader should express and operationalize safety concerns. Safety leadership can also represent a process to attain the desired conditions and prepare the line-up to be engaged and succeed in strategic efforts to promote the importance of safety (Muhammadiyah, 2019). Safety leadership is generally recognized as an essential element of business success, but poor safety leadership on the contrary can hinder the ability of an organization to achieve strategic goals. Meanwhile, according to Cooper (2015), safety leadership is the process of team formation leaders, ensuring teams are involved in fostering security values and preserving the team to achieve organizational safety goals. In addition to focusing on individuals and teams, this type of leadership style also maintains the integrity of the machines and technology used to meet operational procedures standards so that the desired safety objectives can be fully achieved. Ineffective leadership in safety frequently arises from uncertainty about the company's security management program and associated policies. This leaves leaders confused about their obligations to incorporate changes and flexibility (Cooper, 2015). Based on the above hypotheses, it can be summarized that safety leadership is a leadership style that affects and encourages subordinates to carry out activities that emphasize safety values both for themselves and for the organization that ultimately aims to reduce the occurrence of accidents at work.

### 2.3 Safety communication

The objective of the communication is to convey information within the organization, so that the information recipient can clearly understand what the communicator means, in particular what actions the organization expects to take. Communication is a critical component of every human-engaged program. No real, coherent operation can be effectively carried out without effective communication (Zamani et al., 2020). As long as cooperation between team members is strengthened, there will definitely be less friction, which in turn points to more success at work. Safety communication is the extent to which safety information is exchanged openly (Amponsah-Tawaih and Adu, 2016). This may also refer to the process of transferring and sharing security knowledge among members in order to complete their duties safely or to get information about risks (Zamani et al., 2020).

Communication on safety can be formal or informal; formal communication relates to the sharing of knowledge conducted across specified channels; it involves formal communications with supervisors in the form of security instruction, work order, written instructions, protection signs, and toolbox discussions. On the other hand, informal communication is established among working group members, and does not have a structured basis. Informal interactions may in the form of advice, informal conversations or social media use. In short, Huang et al. (2018) stated that safety communication is to effectively communicate about safety to obtain a better knowledge of safety behaviour and the risks of unsafe behaviour. Communication of safety can be used to identify or monitor hazardous situation prior to the incidents. Based on the above definitions, safety communication can be synthesized as cross-functional communication that focuses on work safety practices that involve protection, handling, and future workplace accidents to minimize risks to organizations and individual employees.

### 2.4 Safety commitment

This concept was interpreted as five aspects of management actions by the study conducted by Fruhen et al. (2019) in measuring safety commitment. The five dimensions consist of decision taking, strategy for managers, constructive engagement, and contact with the staff by managers. According to Fruhen, the commitment to safety is considered to be one essential aspect in organizational safety in the literature. Specifically, it is one major element of safety climate or safety culture. The issue of safety commitment appears in studies of ineffective leadership, focusing on senior managerial roles in workplace accidents. Furthermore, the viewpoint of social information processing suggests that individuals understand their work environment through processing social information, and the concept of safety commitment to management is possibly embedded in behaviour that can be seen from leaders (Xue et al., 2020). Behaviour in which a person communicates a commitment is usually linked to the purpose of his/her commitment. Based on the above theories, it can be synthesized that safety commitment is a reflection of work safety commitment with regard to work safety behaviour carried out by the organization as measured by workers' perceptions of all efforts made by management on work safety.

### 2.5 Safety climate

The term safety climate was first proposed by Oah et al. (2018) to explain employees' ideas on the role of safety in organizations. Safety climate represents common opinions among members regarding the organizations' social units, rules, procedures and performs associated with safety (Schwatka and Rosecrance, 2016). The study considered that this perception is reflective of: 1) the priority level of safety in addition to the objectives of organizations, 2) how many safety policies adopted have been applied, 3) the steadiness of safety practices, and 4) the management's commitment to safety. In addition, Xue et al. (2020) argued that improved safety climate of a company, will foster safety performance, and reduce unsafe behaviour among employees. Moreover, safety climate refers to workers' common beliefs on the value of safe behaviour in their jobs. Employees who consider their working environment safe tend to experience fewer accidents than those who regard their working environment dangerous. Additionally, employee perceptions about safety climate can help companies recognize elements that need improvement. Zamani et al. (2020) also argued that safety climate is a guide for workers to adjust their actions to the environment at work. Positive safety climate is created when interactions between the organization and the project team are guided in a way that the organization ensures safe project implementation, provides appropriate and newest personal protective equipment (PPE), and considers safety as their top priority. Based on these explanations, it can be said that safety climate is the result of observations and experiences related to policies, practices and procedures which further shape behaviours that prioritize the safety value in the company.

## 3 Literature review

### 3.1 Safety leadership to safety climate

Recent research in different industries, including construction, has demonstrated a relationship among safety leadership, safety climate (i.e., how employees see organizational commitment to workplace safety) and other safety result (Schwatka et al., 2019). Leaders assistance will provide knowledge of safety training and accident prevention, and help staff develop their safety skills. It is especially necessary to foster safety climate, as it takes time to internalize and articulate the understanding (Schwatka et al., 2019).

Oah et al. (2018) argued that security leadership is an interaction through which leaders may force their members to accomplish organizational safety objectives based on the organization circumstances and individual factors. Also confirmed by claims by Eliyana, Ma'arif, & Muzakki (2019) that optimum job output of employees can only be achieved if the employee's performance is optimised. So, it can be assumed that the actions of managers or members reflects and operationalizes a concern for employee safety. Employees prefer to commit to safety when they have a strong relationship with their boss and manager and maintain open contact about it. So, the idea of safety climate can be well used to explain employee attitudes about the importance and role of protection in the organization.

Safety leaders guide followers to attain the safety goals of the organization in order to decrease the number of accidents at work. his style of leader provides creative thinking and rewards for safety-related achievements. The stimulation forms a working environment that is concerned with safety-related policies, procedures, and practices so that individuals within the organization focus on improving safety in every work they do, from which safety climate is formed. It can be stated that the greater the influence of the leader on safety, the higher the organizational safety climate. Furthermore Muhammadiyah (2019) also found that safety climate is positively influenced by safety leadership. Thus, the hypothesis can be derived as follows:

H1 Safety Leadership has a significant positive effect on Safety Climate.

### 3.2 Safety communication to safety climate

According to Huang et al. (2018) any observed result of regulating the climate of safety at the organizational level is induced by communication of surveillance. Managers who consistently communicate about safety may build better understanding of safe behaviour and possible unsafe behavioural outcomes among employees. It is also recognized that some old safety climate models consider safety communication as a part of safety climate. However, on the other hand, safety communication is theoretically unlike safety climate, it includes organizational rules and performs that can influence or be affected by the safety climate. This guides more work to assume positive views on safety climate related to improved safety communication.

Safety climate has been mostly studied in the construction industry regardless of the safety communication (Zamani et al., 2020). This study considers safety communication and safety climate as different organizational factors for determining the cause of accidents and also as a basis for developing knowledge systems based on negative experiences. The study assumes that safety climate can help implement lessons from past incidents through safety communication. Based on above-mentioned studies, the hypothesis is structured as follows:

H2 Safety Communication has a significant positive effect on Safety Climate.

### 3.3 Safety commitment to safety climate

Stackhouse & Turner (2019) defines safety commitment as the extent to which risky actions are physically avoided, procedures are adhered to and organization's security program is believed. More specifically, it is suggested that the safety commitment strengthens the relationship among safety climate perceptions that can strengthen the tangible commitment of an organization to safety. If the attitude to safety is high, staffs tend to take constructive measures to work carefully and find a better work setting (i.e., assuming that their colleagues are doing the same). So that the perception of safety commitment can then strengthen or reduce the indirect relationship to safety climate.

According to Fruhen et al. (2019) employees may describe the extent to which management displays safety commitment as they consider it as the key factor of safety climate. High safety commitment on management is a proven measure that the company has a positive safety climate. Thus, it can be concluded that a positive safety climate may lead to better safety results such as higher compliance with regulations and fewer injuries at work. From the above explanations, the fourth hypothesis of this study is structured as follows:

H3 Safety Commitment has a significant positive effect on Safety Climate.

### 3.4 Safety leadership to safety behaviour

Xue et al. (2020) proved that effective safety leadership (e.g. visits, feedback) can greatly decrease employee anxiety about workplace accidents and injuries, which means they receive extra confidence to be in a free workplace accident in the future. Safety leadership is considered as a top-level obligation, and the relationship between safety leadership and employee safety behaviour has been investigated in several studies. Furthermore, determining the connection between dimensions of safety leadership and behaviours can guide senior managers to know how to improve their safety leadership, thus helping to decrease the number of accidents and reach wide-ranging safety and health. So, if one aspect of safety leadership is proven to affect safety behaviour, it would be rational to channel resources towards improving that aspect. However, if all dimensions make the same contribution, then a comprehensive approach to development is needed.

A good leader will empower employees and fully enrich them with experience, skills, and knowledge (Lee et al., 2019). This is supported by a statement from Ngadiman et al. (2013) that leadership is the basis of organizational development, because without good leadership, it will be hard to realize organizational goals and to adapt to changes that occur, both inside and outside the organization. The rapid and wide-ranging information distribution is expected to reduce the time needed to learn, enable employees to immediately implement the knowledge, and accelerate the organization's progress towards safety goals. Based on the description above, the hypothesis can be derived as follows:

H4 Safety Leadership has a significant positive effect on Safety Behaviour.

### 3.5 Safety communication to safety behaviour

Communication is the overall experience of the employee exchanging information, thoughts and feelings among leaders, and members, in order to efficiently and productively incorporate technical knowledge and information among teams. Communication is an essential determinant for organizational safety climate, people's mental state, and safety behaviour (He et al., 2019). Safety behaviour is safety related activities conducted within an entity by individuals. Safety behaviour as a primary measure of safety performance has many benefits over lagging metrics, such as injury and death. Safety behaviour data tend to have a normal distribution, so it is easier to interpret the relationship with the antecedents, more reliable, and more useful for safety assessment and intervention. The ability to communicate effectively in a personal and socially appropriate manner determine the impact of safety communication. Therefore, according to He et al. (2019) communication skills can be used to interpret the variance in safety behaviour that results from reasoning, feeling, intrinsic motivation and circumstances.

The literature implies that safety behaviour (e.g., performance) may affect further results such as accidents or injuries, which eventually suggests that safety behaviour can impact safety communication with injury outcomes (Huang et al., 2018). Based on previous findings, the fifth hypothesis of this study will be arranged as follows:

H5 Safety Communication has a significant positive effect on Safety Behaviour.

### 3.6 Safety commitment to safety behaviour

According to Ye et al. (2020) perceived safety commitment as a benchmark through which employees develop information about safety behaviour requirements and success in meeting safety standards. The management's role is building internalized values and beliefs among employees, and giving a clear picture of what goals they should pursue, as well as the type of safety behaviour they are required to achieve. The implementation of safety commitment can be in the form of highly appreciation of safety officers and the setting up of a training program. By witnessing and experiencing these management-friendly processes, structures and procedures, employees strengthen their awareness of the key tasks and obligations that they must perform, including knowledge of safety issues and the value of providing protection.

The theory of organizational commitment suggests that people can undergo different types of safety commitment and this interaction is a psychological condition that determines how they enforce their commitment (Fruhen et al., 2019). The commitments to maintain safe conduct can be defined as core safety activities that individuals need to ensure safety at work. Whereas safety participation behaviour can be seen as activity that does not specifically lead to workplace safety, but it helps to create a work atmosphere that promotes safety. Amponsah-Tawaih & Adu (2016) stated that safety behaviour is one of the major concerns of most organizations worldwide. Based on the findings and explanations from some of the previous studies, the sixth hypothesis of this study can be derived as follows:

H6 Safety Commitment has a significant positive effect on Safety Behaviour.

### 3.7 Safety climate to safety behaviour

According to Ji et al. (2019) safety climate has a tremendous effect on attitudes, values, and behaviour associated with safety in the organization. As an organizational variable, the term "safety climate" refers to common perceptions about policies, procedures, and organizational safety practices, in the work setting. Therefore, safety climate has a significant effect on numerous individual work-related outcomes including safety performance, personal attitudes, well-being and safety-related results. Likewise, employee safety behaviour is closely related to relevant company safety climate. When the level of safety climate changes (strengthening or weakening), then the personality of employees displayed in safety behaviour also changes.

Referring to Social Identity Theory according to Martiny & Rubin (2016), explains that individual's self-concept partly stems from the his/her association to a specific social group, which is accompanied by values, emotions, level of involvement, caring and also a sense of pride in his membership in the group. Referring to the theory, when individuals are in work groups that prioritize work safety values, individuals will feel involved and care for their groups, which in turn will also apply behaviours that prioritize work safety in every job performed because they feel involved for can realize what is considered important by the group.

Safety climate represents various processes that occur within an organization over a specified period of time (Murphy et al., 2019). Thus, there is a constant adjustment of the understanding where workers continuously gather and coordinate safety information as a priority within their company, which is often known to affect the safety behaviour at work. Based on the theory and some previous research above, the following hypotheses can be derived:

H7 Safety Climate has a significant positive effect on Safety Behaviour.

### 3.8 Safety leadership to safety behaviour mediated by safety climate

The implementation of safety leadership approaches and techniques by leaders can help the human resource department by promoting safety and improving the results of safety behaviour (Smith et al., 2016). Such approaches can foster the improvement of employees’ perception toward positive safety climate and initiate, maintain and/or improve agreement with safety behaviour and contribution to safety behaviour. Oah et al. (2018) stated that safety leadership can be a crucial factor in lowering the perceived risk level among employees. Related to the previous statement, according to Smith et al. (2016) suitable leadership style can directly exert good control on subordinates, and ultimately create positive changes that are influenced in perceptions of safety climate. In addition to reducing the level of risk, leaders can create positive changes in safety behaviour. Thus, it is stated that a positive perception of safety leadership will be associated with a positive perception of safety climate, which will be positively related to safety behaviour.

In another study by Martínez-Córcoles et al. (2011), safety climate positively and significantly mediated the influence of leaders on employee safety behaviour. Leaders who underline the importance of safety will increase safety climate which in turn cause an increase in employee participation in safety so that employees will apply safety behaviour. Based on the theory and previous research above, it can be derived as follows:

H8 Safety Climate significantly mediates the effect of Safety Leadership on Safety Behaviour.

### 3.9 Safety communication to safety behaviour mediated by safety climate

According to Ji et al. (2019) An employee's safety behaviour is strongly related to safety climate which affects relevant work. It can be stated here that positive safety climate seems to affect the results of management activities carried out with approaches that lead to safety behaviour. Furthermore Huang et al. (2018) consider safety communication and safety climate as separate main organizational factors that are useful for investigating the reasons of construction accidents and serving as a basis for creating learning systems for reflecting on past incidents. In addition, it is known that safety communication can reduce unsafe behaviour.

From the three findings, it can be concluded that there is a pattern about mediating safety climate to the connection between safety communication and safety behaviour, where effective safety communication originating from management will influence actions and concerns in terms of work safety due to the support of conditions that help it to occur. Safety climate will indirectly promote a more open and free exchange of issues related to safety which in turn will shape behaviours of security concern in the workplace. Based on the findings and explanations from some of the previous studies above, it can be derived from the ninth hypothesis as follows:

H9 Safety Climate significantly mediates the effect of Safety Communication on Safety Behaviour.

### 3.10 Safety commitment to safety behaviour mediated by safety climate

Management with high safety commitment is known as the indicator of positive safety climate in an organization (Fruhen et al., 2019). Positive safety climate results in improved safety outcomes such as compliance with regulations and fewer work accidents. The literature focus of safety commitment is somewhat separated from the conceptualization of commitments in other domains which mainly focus on individual experiences of commitment, and shows that the dimensions of commitment can influence behaviour and outcomes as well as safety behaviour.

The opinion of Ye et al. (2020) that safety commitment is a benchmark in which workers develop cues about expectations for safety behaviour. Such conceptualizations underpinning Safety Commitment will in turn foster workers' concern about the importance of safety procedures and the potential negative impacts that will be demonstrated in behaviour at work. In Ji et al. (2019) and Murphy et al. (2019) prove that safety climate influences Safety Behaviour. It can be seen that a good safety climate in the work environment can produce individual safety behaviour in the organization, because when an organization already has a good level of Safety climate, work safety within the organization has become a priority.

**Figure 1:**
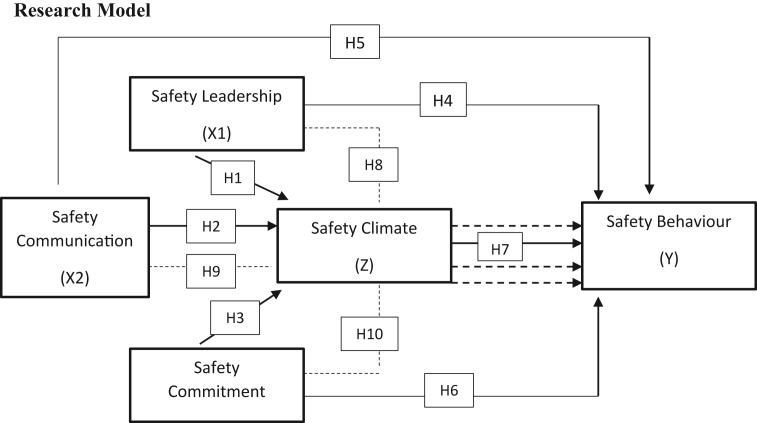
Conceptual framework.

From the explanation above, the effectiveness of safety commitment to safety behaviour will be determined through the conditions of safety climate, where individual organizations have an awareness of work safety. From various research results and explanations above, the tenth hypothesis of this study is as follows (see Figure 1):

H10 Safety Climate significantly mediates the effect of the Safety Commitment on Safety Behaviour.

## 4 Methods

### 4.1 Research time and place

The research was done at PT GMF AeroAsia Tbk in Cengkareng home base and multibase in Kalimantan, Bali & Nusa Tenggara, Sumatra, Sulawesi & East, and Java. The research is expected to be completed within one year, carried out from the beginning of September 2019 to the end of March 2020.

### 4.2 Population and sample

The population is 2,400 employees of PT GMF AeroAsia Tbk and is spread across; Homebase: Cengkareng (9 Departments), Multibase: Kalimantan, Bali & Nusa Tenggara, Sumatra, Sulawesi & East, & Java. While the study sample was 342 respondents based on the Slovin Formula: n = N/(1 + (N x e^2^). Furthermore, informed consent was obtained from all participants in this study.

### 4.3 Data analysis technique

This research uses a quantitative approach, which means the stages are, stating hypotheses and theories, building an analytical model, defining variables, collecting data (primary and secondary), then finally analyzing the research results. This study conducted a test analysis using LISREL 8.8 software (part of Structural Equation Modeling (SEM)) for the data that has been obtained. Structural Equation Modeling (SEM) is a multivariate analysis which is commonly used to analyze complex relationships between variables. Data analysis using SEM can help to thoroughly explain the relationship between variables in the study. SEM is also used to inspect and maintain predefined research models. Meanwhile, LISREL is a statistical software that will be used in structural equation modeling to produce statistical analysis results and factor analysis calculations. LISREL 8.8 software has its own advantages, such as the ability to recognize complex relationships between variables. However, LISREL 8.8 also has the disadvantage of not being able to process SEM data from a small sample size (Hair et al., 1995).

The ethical approval for this research was given by the research and development divison of Universitas Airlangga. This division is represented by Development and Innovation Institute for Publishing Journal and Intellectual Property Rights (LIPJIPHKI), an internal institute which is responsible to supervise Publications and Journals, Innovation and Intellectual Property Rights, as well as Publishing/Universitas Airlangga Press. It is also responsible for developing research and directing the results of innovative research products for the benefit of the community. This institute has the authority to give ethical approval for research done by lecturers’ of Universitas Airlangga or research which are taken place in Universitas Airlangga.

#### 4.3.1 Validity reliability

Goodness of Fit Item statement can be said to be VALID if it meets the requirements: r count > r table (r table = 0.30, N = 30). Meanwhile, a statement can be categorized as RELIABEL if it has a value: Cronchbach Alpha> 0.70.

#### 4.3.2 Confirmatory factor

CFA Test

Significant Critical Ratio (CR) value is > 1.96

A significant P-value is in the range p = 0.001 ≤ x ≤ p = 5% (see Table 1)

**Table 1:** Goodness of fit.

Goodness of Fit Index	Cut of value
Probability	≥0,05
RMSEA	≤0,08
GFI	≥0,90
AGFI	≥0,90
TLI	≥0,95
CFI	≥0,94

#### 4.3.3 Hypothesis & model

Hypothesis Test is testing a hypothesis where all variables must have T-Values < T-Stat. While the Structural Model is to find out the SEM model equation formula, so the coefficients of the proposed model equation can be obtained.

### 4.4 Operational definition

#### 4.4.1 Safety behaviour

Safety Behaviour is defined as employee behaviour in terms of workplace safety that is reflected through individual actions that contribute to building a good work safety environment. The indicators include safety compliance and safety participation. The indicators used in measuring safety behavior in this study refer to research by Lu and Yang (2010).

#### 4.4.2 Safety leadership

Safety Leadership is a leadership style that will influence and invite subordinates to carry out activities that prioritize safety values both for themselves and the company which can minimize incident work accidents. The indicators include safety motivation, safety policies, and safety concerns. The indicators used in measuring safety leadership in this study refer to research by Lu &Yang (2010).

#### 4.4.3 Safety communication

Safety Communication refers to cross-functional communication with a focus on work safety procedures that includes safety, handling, and potential workplace incidents to avoid events that have a negative impact on the organization or individual workers. The indicators include comfort when discussing safety issues with Supervisors, the belief that their Supervisors openly accept ideas to improve safety, and the belief that their Supervisors encourage open communication about safety. The indicators used in measuring safety communication in this study refer to research by Alsamadani et al. (2015).

#### 4.4.4 Safety commitment

Safety Communication is a reflection of safety commitments on work safety behaviours carried out by the organization with a measure of workers' assumptions about all efforts made by management on work safety, with indicators that include greater management commitment, better perception of safety rules and procedures, and greater integration of safety levels. The indicator used in measuring safety commitment in this study refers to the research by Fruhen et al. (2019).

#### 4.4.5 Safety climate

Safety Climate is related to reflections on work safety priorities relating to rules, performs and trials that subsequently form behaviours that prioritize the value of work safety within the company, with indicators that include knowledge, skills, abilities, intelligence, motives, and personality. The indicator used in measuring safety climate in this study refers to the research of Wu et al. (2008).

## 5 Results

Based on 342 questionnaires to the employees of PT GMF AeroAsia Tbk, the respondent profile in this study was distinguished by sex, age, last education, working period, and position. The following are the results of the respondents' profile analysis (see Table 2):

**Table 2:** Respondent profile.

**I. Gender**		
Gender	Numbers	Percentage (%)
Male	288	84
Female	54	16
**II. Age**		
Age	Numbers	Percentage (%)
<21	3	1
21–30	137	40
30–40	37	11
41–50	81	24
>50	84	25
**III. Education**		
Education	Numbers	Percentage (%)
Senior High School	116	34
D2	22	6
D3	65	19
S1	114	33
S2	25	7
**IV. Working Period**		
Period	Numbers	Percentage (%)
1–5	125	37
6–10	65	19
>10	152	44
**V. Position**		
Position	Numbers	Percentage (%)
Staff	10	3
Purchaser	12	4
Specialist	18	5
General Manager & equals	18	5
Planner	29	8
Manager	34	10
Inspector	36	11

Testing Instrument N = 30 and r-table = 0.30 Statement items can be said to be valid if they meet the requirements: r count > r table. All indicators are VALID, because they have: r count> 0.30 (see Tables 3 and 4).

**Table 3:** Test validity of instruments.

Indicator	r count	Note	Indicator	r count	Note
SB1	0.480	Valid	SCC1	0.857	Valid
SB2	0.383	Valid	SCC2	0.857	Valid
SB3	0.532	Valid	SCC3	0.651	Valid
SB4	0.440	Valid	SCC4	0.910	Valid
SB5	0.668	Valid	SCC5	0.752	Valid
SB6	0.588	Valid	SCC6	0.702	Valid
SL1	0.545	Valid	SCC7	0.870	Valid
SL2	0.745	Valid	SCM1	0.784	Valid
SL3	0.825	Valid	SCM2	0.806	Valid
SL4	0.627	Valid	SCM3	0.799	Valid
SL5	0.885	Valid	SCM4	0.891	Valid
SL6	0.855	Valid	SCM5	0.891	Valid
SL7	0.746	Valid	SCM6	0.691	Valid
SL8	0.828	Valid	SCM7	0.781	Valid
SL9	0.614	Valid	SCM8	0.820	Valid
SL10	0.725	Valid	SCL2	0.868	Valid
SL11	0.826	Valid	SCL3	0.793	Valid
SL12	0.750	Valid	SCL4	0.189	Valid
SL13	0.803	Valid	SCL5	0.771	Valid
SL14	0.762	Valid	SCL6	0.678	Valid
SL15	0.828	Valid	SCL7	0.635	Valid
SL16	0.790	Valid	SCL8	0.454	Valid
			SCL9	0.876	Valid
			SCL10	0.806	Valid

**Table 4:** Instrument reliability test.

Variables	Cronbach's Alpha	N of Items
Safety Behaviour	.758	6
Safety Leadership	.958	16
Safety Communication	.935	7
Safety Commitment	.946	8
Safety Climate	.912	10

Testing CFA (Confirmatory Factor Analysis). Number of samples 342 or 250 < x 0.35. Statement items can be said to be valid if they meet the requirements: Loading factor> 0.35. All indicators are valid, because they have: Loading factor> 0.35 (see Table 5).

**Table 5:** Stage 1 CFA test.

Indicator	Loading Factor	Note	Indicator	Loading Factor	Note
SB1	0.820	Valid	SCC1	0.905	Valid
SB2	0.842	Valid	SCC2	0.889	Valid
SB3	0.869	Valid	SCC3	0.902	Valid
SB4	0.747	Valid	SCC4	0.889	Valid
SB5	0.579	Valid	SCC5	0.883	Valid
SB6	0.649	Valid	SCC6	0.874	Valid
SL1	0.734	Valid	SCC7	0.873	Valid
SL2	0.789	Valid	SCM1	0.744	Valid
SL3	0.840	Valid	SCM2	0.820	Valid
SL4	0.711	Valid	SCM3	0.822	Valid
SL5	0.764	Valid	SCM4	0.883	Valid
SL6	0.851	Valid	SCM5	0.898	Valid
SL7	0.888	Valid	SCM6	0.787	Valid
SL8	0.875	Valid	SCM7	0.829	Valid
SL9	0.853	Valid	SCM8	0.873	Valid
SL10	0.823	Valid	SCL2	0.833	Valid
SL11	0.889	Valid	SCL3	0.804	Valid
SL12	0.816	Valid	SCL4	0.625	Valid
SL13	0.904	Valid	SCL5	0.699	Valid
SL14	0.894	Valid	SCL6	0.900	Valid
SL15	0.887	Valid	SCL7	0.907	Valid
SL16	0.879	Valid	SCL8	0.843	Valid
			SCL9	0.859	Valid
			SCL10	0.837	Valid

The number of samples is 342 or 250 < x 0.35. Statement items can be said to be valid if they meet the requirements: Loading factor> 0.35. All indicators are valid, because they have: Loading factor> 0.35 (see Table 6).

**Table 6:** Stage 2 CFA test.

Indicator	Loading Factor	Note	Indicator	Loading Factor	Note
SB1	0.860	Valid	SCM5	0.905	Valid
SB2	0.872	Valid	SCM6	0.792	Valid
SB3	0.864	Valid	SCM8	0.875	Valid
SL3	0.831	Valid	SCL1	0.755	Valid
SL11	0.892	Valid	SCL5	0.668	Valid
SL13	0.905	Valid	SCL8	0.865	Valid
SCC2	0.887	Valid	SCL9	0.874	Valid
SCC3	0.900	Valid	SCL10	0.845	Valid
SCC4	0.889	Valid			
SCC6	0.859	Valid			

**Figure 2:**
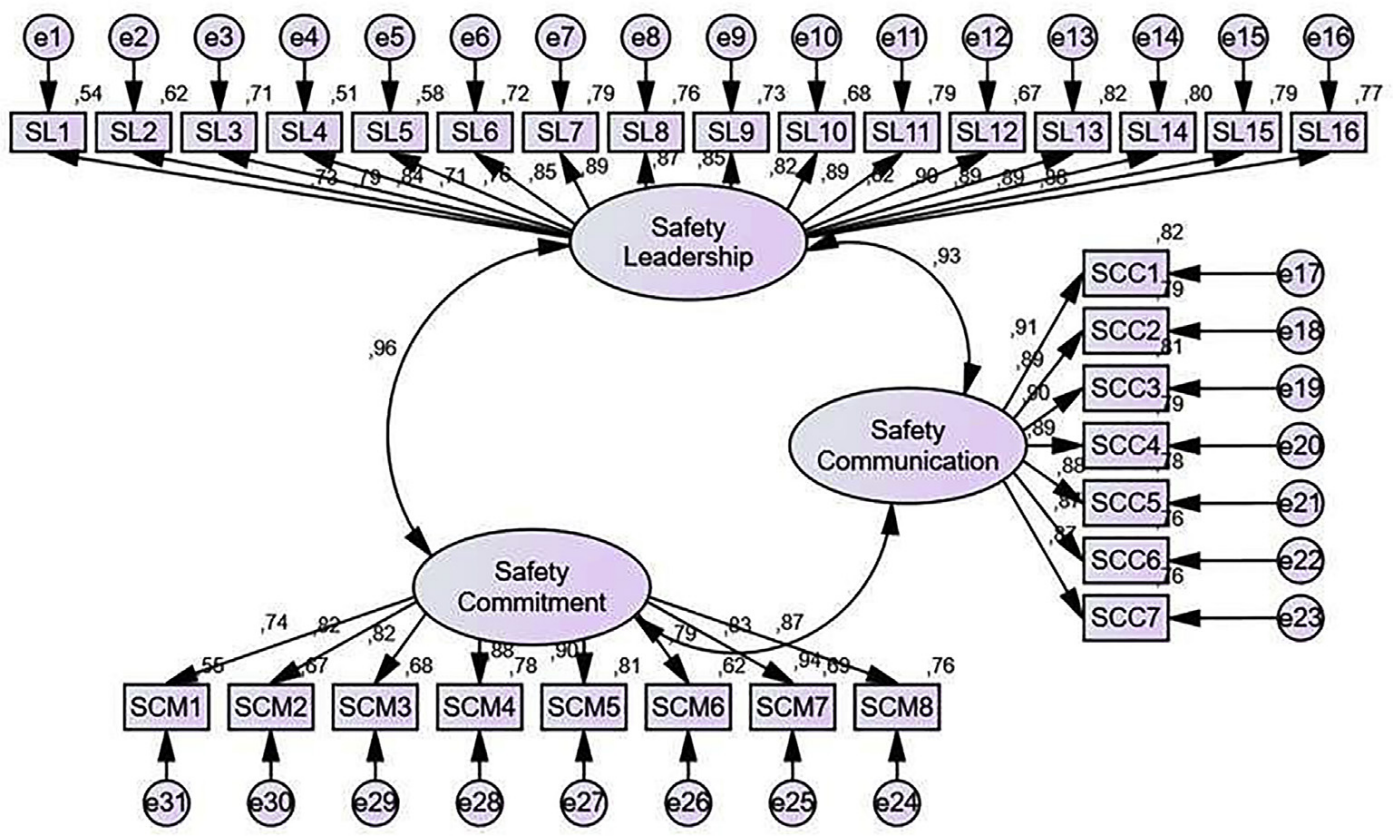
Initial model of exogenous constructions.

Exogenous construct confirmatory test consists of Safety leadership, Safety commitment, and Safety communication variables (see Table 7; Figure 2).

**Table 7:** Results of exogenous constructive output.

Goodness of Fit Index	Model Result	Cut of value	Note
*Probability*	0.000	≥0.05	Not fit
RMSEA	0.084	≤0.08	Not fit
GFI	0.767	≥0.90	Not fit
AGFI	0.732	≥0.90	Not fit
TLI	0.912	≥0.95	Not fit
CFI	0.918	≥0.94	Not fit

**Figure 3:**
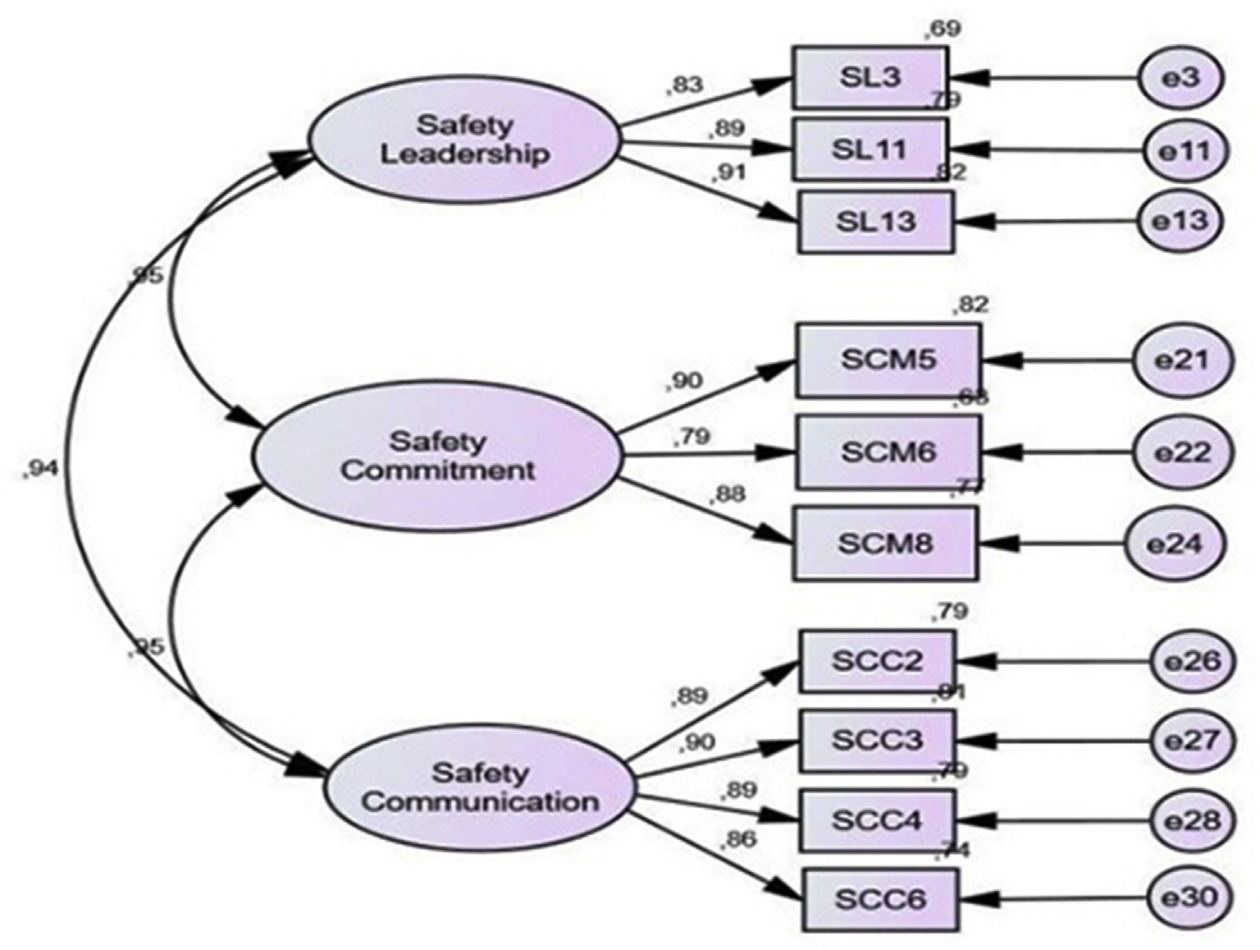
Exogenous construction models after modification.

To produce a smaller Chi-Square and a better model, Modification indices are then carried out (Arbuckle, 2008). Technically this is done by providing several recommendations for adding links/connections that can reduce the chi-square value so that the model becomes fitter. Modification of the model is done by considering the greatest value of Modification Indexes (MI) available on AMOS 22 software (see Table 8; Figure 3).

**Table 8:** Results of exogenous constructions after modification.

Goodness of Fit Index	Model Result	Cut of value	Note
*Probability*	0.079	≥0.05	Fit
RMSEA	0.033	≤0.08	Fit
GFI	0.976	≥0.90	Fit
AGFI	0.959	≥0.90	Fit
TLI	0.995	≥0.95	Fit
CFI	0.997	≥0.94	Fit

**Figure 4:**
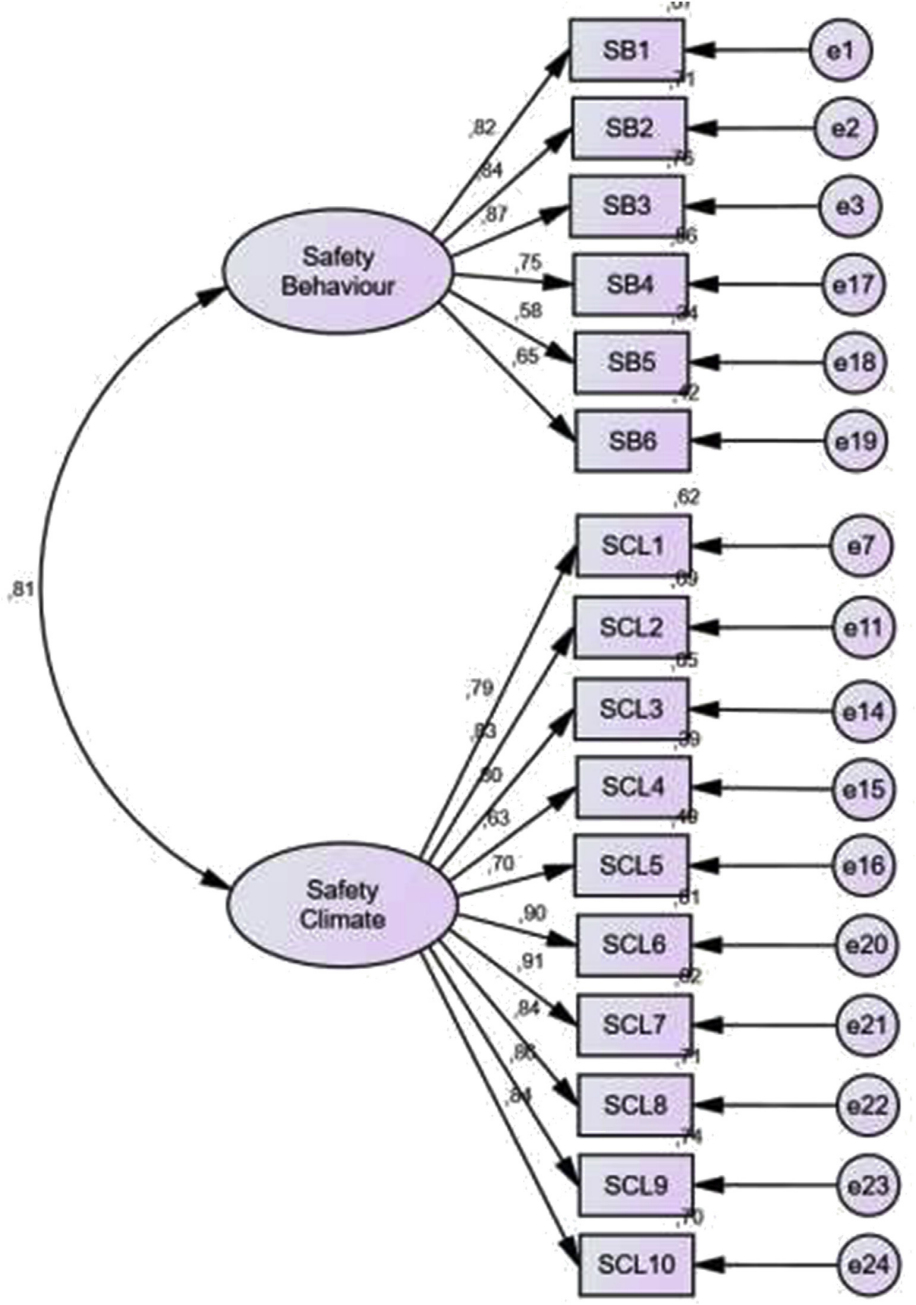
Initial model of endogenous constructions.

Endogenous Constructive Confirmatory Test consists of Safety climate and Safety behaviour variables (see Table 9; Figure 4).

**Table 9:** Results of endogenous constructive output.

Goodness of Fit Index	Model Result	Cut of value	Note
*Probability*	0.000	≥0.05	Not fit
RMSEA	0.142	≤0.08	Not fit
GFI	0.747	≥0.90	Not fit
AGFI	0.665	≥0.90	Not fit
TLI	0.831	≥0.95	Not fit
CFI	0.855	≥0.94	Not fit

**Figure 5:**
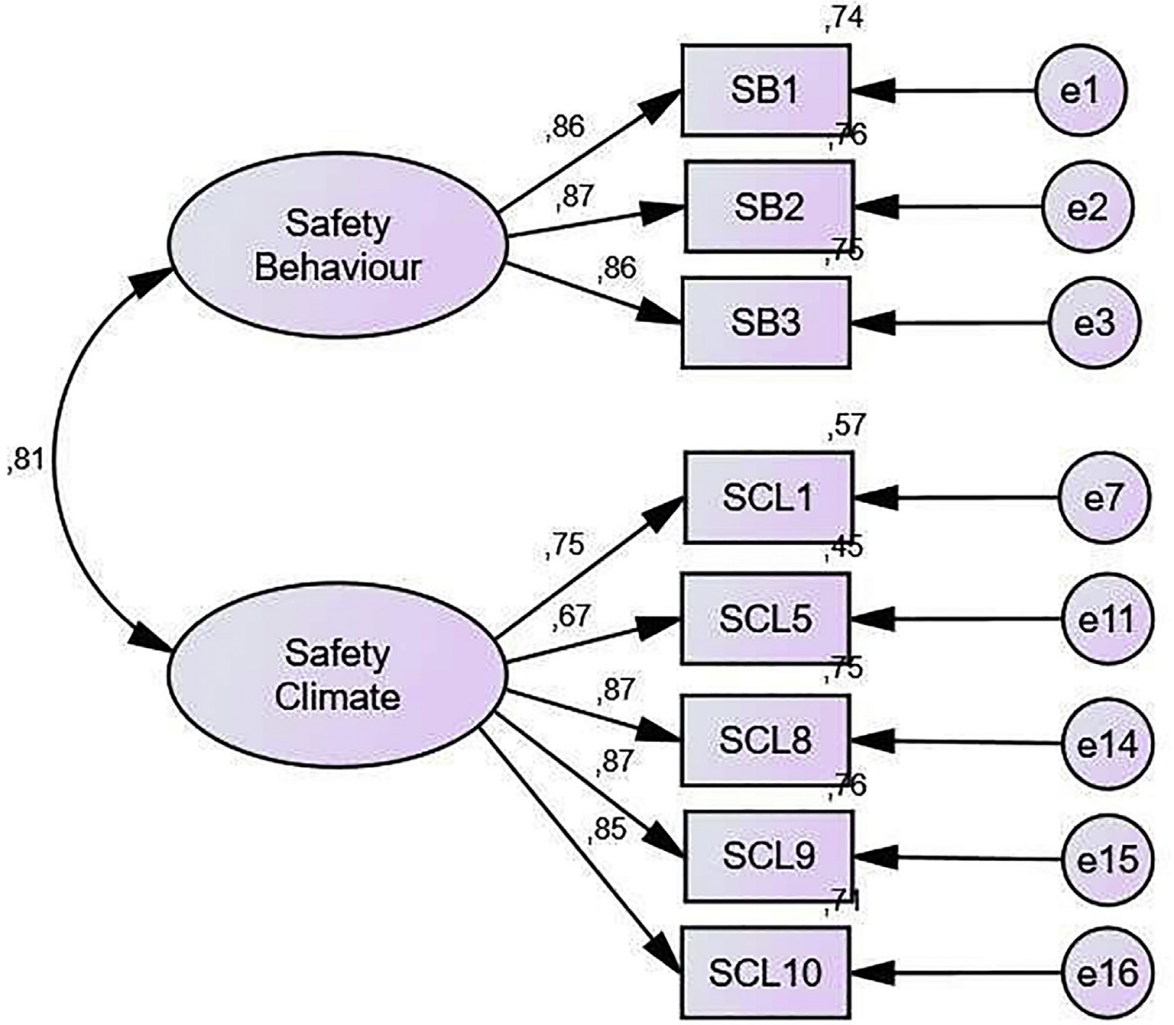
Endogenous construction models after modification.

To produce a smaller Chi-Square and a better model, Modification indices are then carried out (Arbuckle, 2008). Technically this is done by providing several recommendations for adding links/connections that can reduce the chi-square value so that the model becomes fitter. Modification of the model is done by considering the greatest value of Modification Indexes (MI) available on AMOS 22 software (see Table 10; Figure 5).

**Table 10:** Results of endogenous constructions after modification.

Goodness of Fit Index	Model Result	Cut of value	Note
*Probability*	0.128	≥0.05	Fit
RMSEA	0.033	≤0.08	Fit
GFI	0.982	≥0.90	Fit
AGFI	0.966	≥0.90	Fit
TLI	0.995	≥0.95	Fit
CFI	0.996	≥0.94	Fit

### 5.1 Structural equation model (SEM) testing

**Figure 6:**
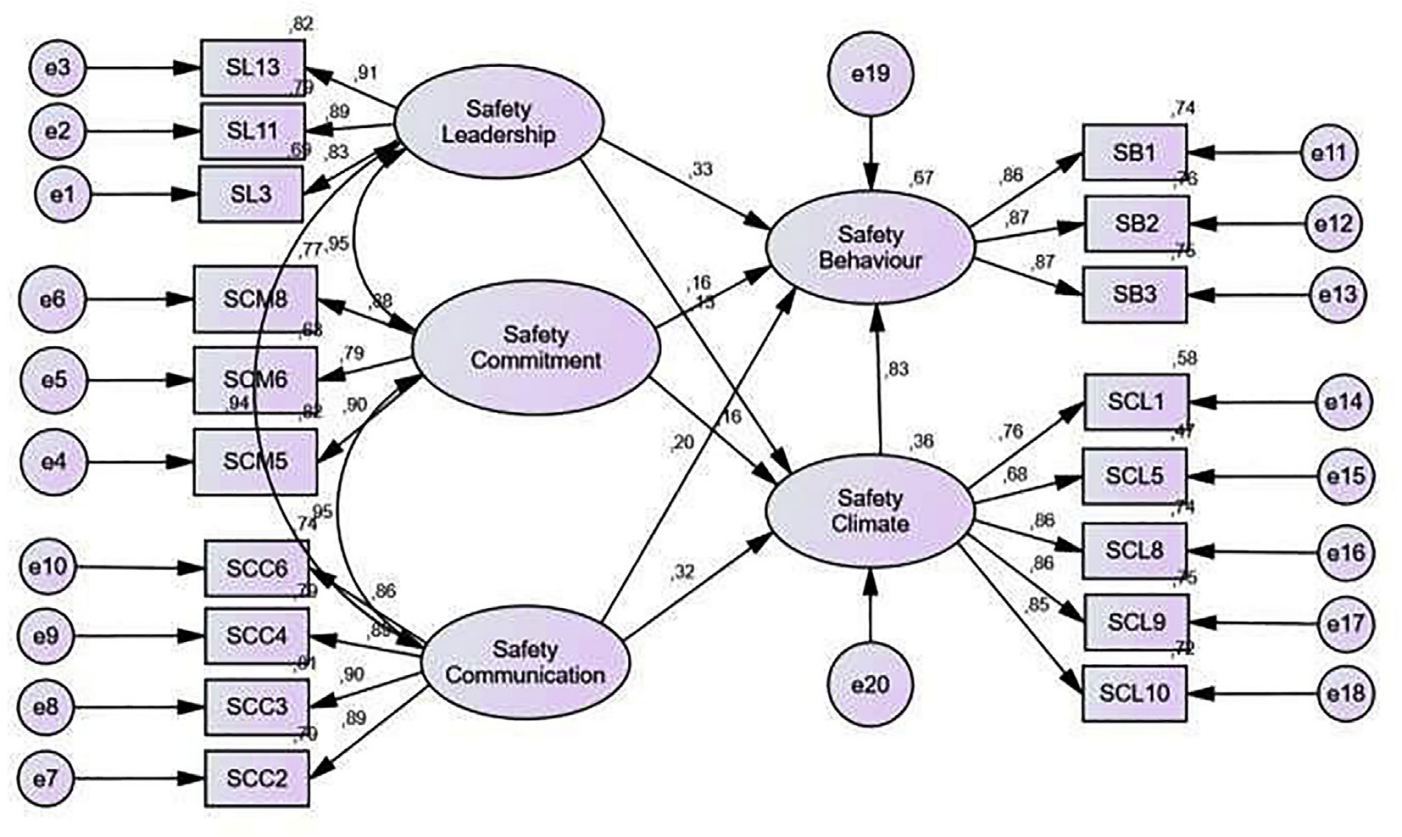
Combined models.

Results of Initial Combined Model Outputs can be seen in Table 11 and are illustrated in Figure 6, which explains the results of the Goodness of Fit Index. It can be seen that Probability and AGFI show results that are not fit because the resulting result model value does not reach the cut of value. Meanwhile, the values from RMSEA, GFI, TLI, and CFI have shown a Fit value because it has met the research requirements, which is more than the cut of value.

**Figure 7:**
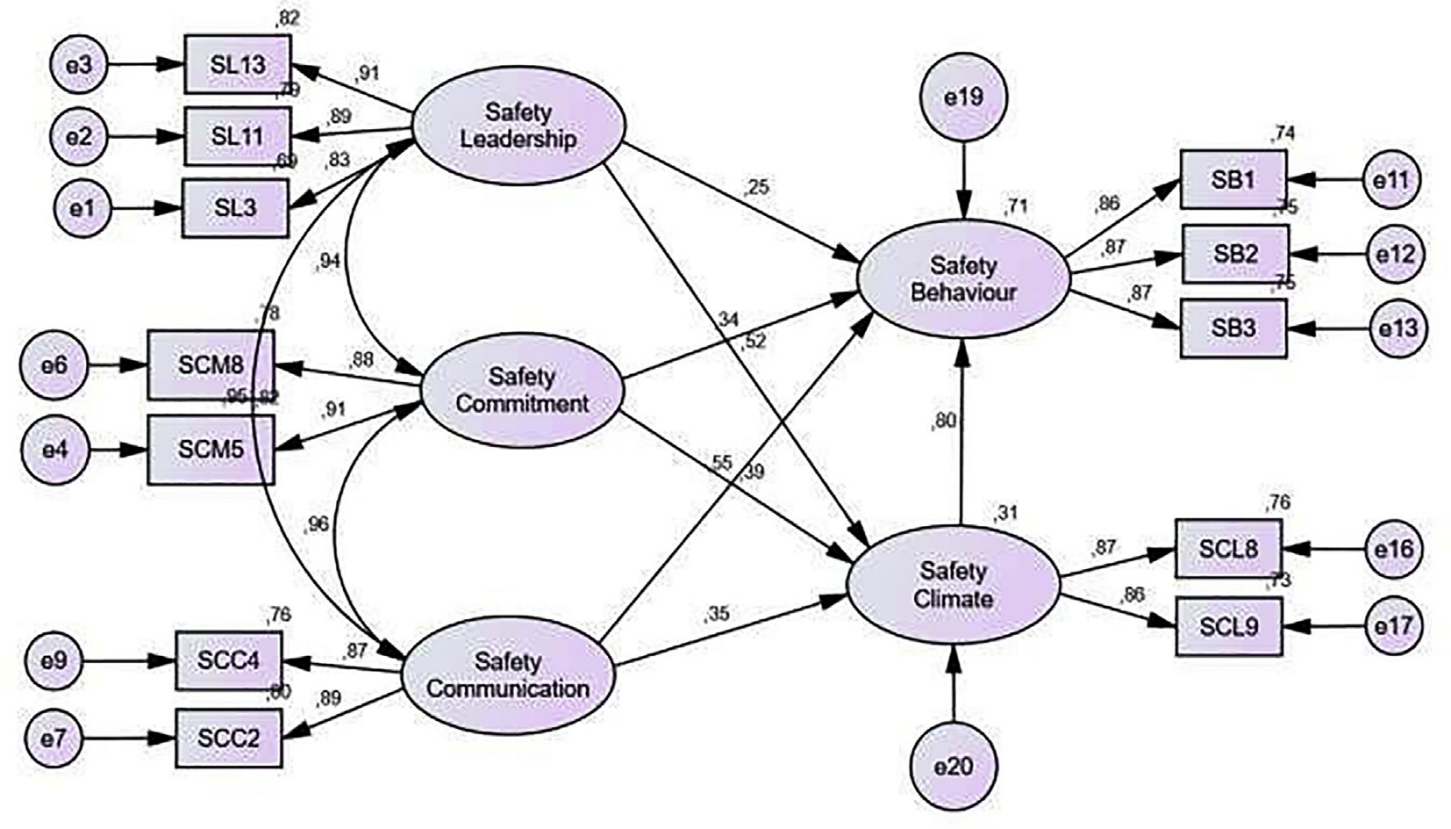
Combined models after modification.

Furthermore, the results of Combined Models After Modification in Table 12 are depicted by Figure 7 which explains the results of the Goodness of Fit Index. These results indicate that Probability, RMSEA, GFI, AGFI, TLI, and CFI have shown a Fit value because they have met the research requirements, namely, the resulting model value reaches or more than the cut of value. Thus, the measurement model that can be seen from the results of the Goodness of Fit Index shows a fairly good fit of the data collected.

**Table 11:** Results of initial combined model outputs.

*Goodness of Fit Index*	Model Result	*Cut of value*	Note
*Probability*	0.000	≥0.05	Not fit
RMSEA	0.058	≤0.08	Fit
GFI	0.922	≥0.90	Fit
AGFI	0.893	≥0.90	Not fit
TLI	0.969	≥0.95	Fit
CFI	0.974	≥0.94	Fit

**Table 12:** Results of combined model output after modification.

Goodness of Fit Index	Model Result	Cut of value	Note
*Probability*	0.206	≥0.05	Fit
RMSEA	0.022	≤0.08	Fit
GFI	0.976	≥0.90	Fit
AGFI	0.957	≥0.90	Fit
TLI	0.997	≥0.95	Fit
CFI	0.998	≥0.94	Fit

### 5.2 Model fit assessment

The output in the Regression Weight Table is used to determine whether the independent variable has a significant effect on the dependent variable. Significant Critical Ratio (CR) value is > 1.96. The significant P-value is a range of p = 0.001 ≤ x ≤ p = 5% (0.05). The symbol ∗∗∗ indicates a value far below 0.001, meaning that the related variable significantly influences (see Table 13).

**Table 13:** Regression weight (output from path analysis).

			Estimate	S.E.	C.R.	P-value
*Safety Leadership*	→	*Safety Climate*	1.025	0.198	5.182	∗∗∗
*Safety Communication*	→	*Safety Climate*	1.471	0.255	5.772	∗∗∗
*Safety Commitment*	→	*Safety Climate*	0.932	0.204	4.560	∗∗∗
*Safety Leadership*	→	*Safety Behaviour*	0.441	0.094	4.685	∗∗∗
*Safety Communication*	→	*Safety Behaviour*	1.055	0.122	8.618	∗∗∗
*Safety Commitment*	→	*Safety Behaviour*	0.620	0.097	6.426	∗∗∗
*Safety Climate*	→	*Safety Behaviour*	0.737	0.025	29.682	∗∗∗

AMOS software does not provide the results of the significance of the indirect effect between the independent variables on the dependent variable through the intervening variable so that a sobel test is necessary.

#### 5.2.1 Safety leadership

$$sab=\;\sqrt{b^{2}sa^{2}+a^{2}sb^{2}+\;sa^{2}sb^{2}\;}$$

$$t=\;\frac{ab}{sab}=\;\frac{\left(1.025\right)\left(0.737\right)}{0.148241}=5.0958$$

Because t count > t table or 5.0958 > 1.96; then the indirect effect is significant or there is mediation. Meaning: Safety Climate mediates the Safety Leadership variable to Safety Behaviour.

#### 5.2.2 Safety communication

$$sab=\;\sqrt{b^{2}sa^{2}+a^{2}sb^{2}+\;sa^{2}sb^{2}\;}$$

$$t=\;\frac{ab}{sab}=\;\frac{\left(0.255\right)\left(0.737\right)}{0.1916}=5.6581$$

Because t count > t table or 5.6581 > 1.96; then the indirect effect is significant/there is mediation. Meaning: Safety Climate mediates the Safety Communication variable to Safety Behaviour.

#### 5.2.3 Safety commitment

$$sab=\;\sqrt{b^{2}sa^{2}+a^{2}sb^{2}+\;sa^{2}sb^{2}\;}$$

$$t=\;\frac{ab}{sab}=\;\frac{\left(0.204\right)\left(0.737\right)}{0.15222}=4.5122$$

Because t count > t table or 4.5122 > 1.96; then the indirect effect is significant or there is mediation. Meaning: Safety Climate mediates the Safety Commitment variable towards Safety Behaviour.

## 6 Discussion

### 6.1 Safety leadership on safety climate

Based on the results of the research analysis, it was found that safety leadership had a positive and significant effect on the safety climate. Thus, it can be said that the higher the safety leadership at PT GMF AeroAsia Tbk is, the higher the safety climate in the company is. This supports the findings of Muhammadiyah (2019) who also found that safety leadership affects safety climate positively. Safety leadership at PT GMF AeroAsia can provide intellectual stimulation and appreciation for achievements related to work safety, which will then form a work environment that is aware of policies, procedures and practices relating to safety. This is what makes employees at PT GMF AeroAsia prioritize safety at work and subsequently will also form a good safety climate.

### 6.2 Safety communication on safety climate

Based on the results of the research analysis, it was found that safety communication had a positive and significant effect on the safety climate. Thus, it can be said that when safety communication at PT GMF AeroAsia Tbk increases, the safety climate in the company also increases. Safety communication implemented at PT GMF AeroAsia Tbk will create supervisory communication that can make organizational-level safety climate well observed. Supervisors at PT GMF AeroAsia Tbk who communicate effectively about safety, will create a better understanding of safe behavior and the risks of unsafe behavior.

### 6.3 Safety commitment on safety climate

Based on the results of the research analysis, it was found that the safety commitment had a positive and significant effect on the safety climate. Hence, it can be said that an increase in safety commitment at PT GMF AeroAsia Tbk, will be followed by an increase in the safety climate in the company. When the safety commitment at PT GMF AeroAsia Tbk is positive, employees will tend to take active strategies to work safely and perceive that their work environment is safer (for example because they think their colleagues are doing the same). That way, the perception of safety commitment at PT GMF AeroAsia Tbk will be able to strengthen the safety climate.

### 6.4 Safety leadership on safety behavior

Based on the results of the research analysis, it was found that safety leadership had a positive and significant effect on safety behavior. Thus, it can be said that when the safety leadership at PT GMF AeroAsia Tbk increases, the safety behavior in the company also increases. Through safety leadership, the delivery of information becomes effective and comprehensive, thereby reducing the time needed to study, so that employees can immediately apply safety knowledge at work, and accelerate the progress of the organization towards safety goals. This is how the relationship between safety leadership and safety behavior is created at PT GMF AeroAsia Tbk.

### 6.5 Safety communication on safety behavior

Based on the results of the research analysis, it was found that safety communication had a positive and significant effect on safety behavior, which means that the higher the safety communication at PT GMF AeroAsia Tbk, the higher the safety behavior in the company. The impact of safety communication at PT GMF AeroAsia Tbk will cause employee communication about safety to be effective and appropriate, personally and socially. That way, safety communication competence will be the key to safety behavior in creating good safety performance.

### 6.6 Safety commitment on safety behavior

Based on the results of the research analysis, it was found that safety commitment had a positive and significant effect on safety behavior. Hence, it can be said that when the safety commitment increases, the safety behavior will also increase. Management at PT GMF AeroAsia Tbk plays an important role in building values and beliefs that are internalized by employees, through contextual cues obtained from the safety commitment. This is achieved by giving their employees a clear picture of what goals they should strive for, as well as what safety behaviors they should do.

### 6.7 Safety climate on safety behavior

Based on the results of the research analysis, it was found that the safety climate has a positive and significant effect on safety behavior. Thus, it can be said that an increase in the safety climate at PT GMF AeroAsia Tbk will trigger an increase in safety behavior. The safety climate refers to the shared perception of organizational safety policies, procedures and practices in a work environment that has an important impact on employee safety behavior, including safety performance, subjective attitudes, personal welfare, and other safety related outcomes. When the level of safety climate changes (strengthens or weakens), the proactive personality of GMF AeroAsia Tbk employees on safety behavior will also change.

### 6.8 Safety leadership on safety behavior mediated by safety climate

Based on the results of the research analysis, it was found that the effect of safety leadership on safety behavior through the safety climate is positive and significant, or in other words, safety leadership can indirectly influence safety behavior through the safety climate. Therefore, when safety leadership can be implemented properly at PT GMF AeroAsia Tbk, safety behavior will increase, and through a good safety climate, safety behavior will also increase. Safety leadership at PT GMF AeroAsia Tbk plays a role in reducing the level of risk that is felt among employees, and will later create positive changes influenced by perceptions of the safety climate and create positive changes to safety behavior. In addition, this finding is also in line with Martínez-Córcoles et al. (2011) who found that safety climate positively and significantly mediates the influence of leaders on employee safety behavior.

### 6.9 Safety communication on safety behavior mediated by safety climate

Based on the results of the research analysis, it was found that the effect of safety communication on safety behavior through the safety climate is positive and significant, or it can be said that safety communication has an indirect effect on safety behavior mediated by the safety climate. When safety communication is well established at PT GMF AeroAsia Tbk, then safety behavior will increase, and through a good safety climate, safety behavior can also increase. Effective safety communication from management will assist management activities related to safety behavior, namely the actions and concerns of employees in terms of work safety, and support from a positive safety climate will facilitate this process. This supports Huang et al. (2018) who consider safety communication and safety climate as the main organizational determinants for investigating the causes of construction accidents. These factors are also the basis for creating behavior in the form of a learning system to learn from past negative experiences.

### 6.10 Safety commitment on safety behavior mediated by safety climate

Based on the results of the research analysis, it was found that the effect of safety commitment to safety behavior through the safety climate has a positive and significant effect, or it can be said that the safety commitment has an indirect effect on safety behavior through the safety climate. This is in line with the view of Fruhen et al. (2019) that employees will see the extent to which management in terms of safety commitment is a key aspect of their perception of the safety climate that leads to the creation of safety behavior. Therefore, it can be said that when safety commitment can be realized properly at PT GMF AeroAsia Tbk, then safety behavior will be realized, and through a good safety climate, safety behavior will increase. Safety commitment is built through the conceptualization of commitments that focus on individual experiences and is a benchmark for employees to develop signals regarding their expectations of having a positive safety climate in the work environment, which can also result in employee safety behavior.

## 7 Conclusion

Safety leadership has a direct positive effect on safety climate. That is, if safety leadership increases, then safety climate increases. This result means that Senior Managers must trust their employees, because this is an implementation of safety leadership. Managers also need to encourage employees to work more safely which is a reflection of safety climate.

Safety communication has a direct positive effect on safety climate. That is, if safety communication increases, the safety climate will increase. This result means that management must communicate lessons from accidents to improve work safety which is an indicator in reflecting safety communication and the need to encourage to work more safely which is an indicator in reflecting safety climate.

Safety commitment has a direct positive effect on safety climate. That is, increased safety commitment will increase safety climate. This result means that Management must act decisively when work safety issues are raised which are indicators in reflecting safety commitment and the need to encourage work more safely which is an indicator in reflecting safety climate.

Safety leadership has a direct positive effect on safety behaviour. That is, an increase in safety leadership will increase safety behaviour. This result means that Senior Managers must trust their employees as indicators in reflecting safety leadership and emphasize the need to comply with safety rules according to standard operating procedures at GMF as indicators in reflecting safety behaviour.

Safety communication has a direct positive effect on safety behaviour. That is, increased safety communication will increase safety behaviour. This result means that management must communicate lessons learned from accidents to improve work safety as an indicator of safety communication and stress the need to comply with work safety rules according to standard operating procedures at GMF which is an indicator in reflecting safety behaviour.

Safety commitment has a direct positive effect on safety behaviour. That is, increased safety commitment, it will increase safety behaviour. This result means that Management must act decisively when work safety issues are raised which are indicators in reflecting safety commitment and the need to comply with work safety rules according to standard operating procedures at GMF which are indicators in reflecting safety behaviour.

Safety climate has a direct positive effect on safety behaviour. That is, safety climate increases, it will increase safety behaviour. These results mean that GMF must provide flexibility to its employees to take work safety measures which are indicators in reflecting safety climate and the need to comply with work safety rules in accordance with operational standard procedures at GMF which are indicators in reflecting safety behaviour.

Safety leadership has an indirect positive effect on safety behaviour through safety climate. That is, if safety leadership increases, it will increase safety behaviour through safety climate. This result means that companies must pay attention to safety leadership if they want to increase employee safety behaviour through safety climate.

Safety communication has a positive indirect effect on safety behaviour through safety climate. That is, if safety communication increases, it will increase safety behaviour through safety climate. This result means that companies must pay attention to safety communication if they want to increase employee safety behaviour through safety climate.

Safety commitment has a positive indirect effect on safety behaviour through safety climate. That is, if safety commitment increases, it will increase safety behaviour through safety climate. This result means that companies must pay attention to safety commitment if they want to increase employee safety behaviour through safety climate.

## 8 Implications

Safety leadership has a positive direct effect on safety climate and a positive effect both directly and indirectly on safety behaviour. To improve employee safety behaviour and safety climate, it is necessary to increase safety leadership. To improve safety leadership, Senior Managers can form a system of work safety responsibilities, and most importantly must trust their employees as it is the indicator of safety leadership variables that can drive employee safety behaviour.

Safety communication has a positive direct effect on safety climate and a positive effect both directly and indirectly on safety behaviour. To improve employee safety behaviour and safety climate, safety communication is needed. To improve safety communication can be done by encouraging feedback from employees about work safety issues, and most importantly, management must communicate lessons from accidents to improve work safety which is the most important indicator of safety communication variables that can drive employee safety behaviour.

Safety commitment has a positive direct effect on safety climate and a positive effect both directly and indirectly on safety behaviour. To improve employee safety behaviour and safety climate, it is necessary to increase safety commitment. To improve safety commitment, management can discipline employees who are indicated to work unsafe, and the most important thing is that management must act decisively when safety issues are raised, which is the most important indicator of safety commitment variables that can spur employee safety behaviour.

Safety climate has a direct positive effect on safety behaviour. To increase safety behaviour, an increase in safety climate is needed. To improve safety climate can be done by GMF giving flexibility to employees to take safety measures, and the most important thing is to comply with work safety rules according to standard operating procedures at GMF which is the most important indicator on safety climate variables that can spur employee safety behaviour.

## 9 Suggestions

### 9.1 For further research

Future research is expected to examine other independent variables that affect safety climate outside of safety communication, safety leadership, and safety commitment because the suitability parameters of the structural R model are low at 0.407 which means that the variability of safety climate can be explained by the variability of safety communication variables, safety leadership, and safety commitment of 40.7%, while the remaining 59.3% is explained by other variables.

### 9.2 For management of PT GMF AeroAsia Tbk

Companies are advised to pay attention to safety leadership in order to spur safety climate and safety behaviour of employees for example Management team must set a work safety incentive system which is the lowest indicator of safety leadership variables. Next, companies should pay attention to safety communication to create safety climate and safety behaviour of employees for example Management team should conduct campaigns to promote safe work practices which is the lowest indicator on the safety communication variable. Company should next pay attention to safety commitments to build safety climate and safety behaviour of employees meaning that Management should reward employees who apply safety behaviour as the lowest indicator of safety commitment variable. Next, focus should also be given to safety climate in order to create employee's safety behaviour by improving high-quality work safety. Moreover, safety commitment has a positive indirect effect on safety behaviour through safety climate, which means that if safety commitment increases, it will increase safety behaviour through safety climate. This result means that companies must pay attention to safety commitment if they want to increase employee safety behaviour through safety climate.

## Declarations

### Author contribution statement

A. Eliyana: Conceived and designed the experiments; Analyzed and interpreted the data.

E.N. Adi: Performed the experiments; Wrote the paper.

H. Hamidah: Contributed reagents, materials, analysis tools or data.

### Funding statement

This research did not receive any specific grant from funding agencies in the public, commercial, or not-for-profit sectors.

### Data availability statement

Data will be made available on request.

### Declaration of interests statement

The authors declare no conflict of interest.

### Additional information

No additional information is available for this paper.
